# Resilience to climate variation in a spatially structured amphibian population

**DOI:** 10.1038/s41598-018-33111-9

**Published:** 2018-10-02

**Authors:** A. Weinbach, H. Cayuela, O. Grolet, A. Besnard, P. Joly

**Affiliations:** 1 UMR 5023 LEHNA, Université de Lyon, Université Lyon1, CNRS, ENTPE, Campus de la Doua, 69622 Villeurbanne, France; 2 CNRS, PSL Research University, EPHE, UM, SupAgro, IRD, INRA, UMR 5175 CEFE, F-34293 Montpellier, France

## Abstract

Understanding the impact of weather fluctuations on demographic parameters is of crucial interest to biodiversity research in a context of global climate change. Amphibians are valuable candidates for investigating this topic due to their strong physiological dependence on water availability and temperature. In this study, we took advantage of data from a long-term capture–mark–recapture (CMR) monitoring program of a great crested newt (*Triturus cristatus*) population inhabiting a 12-pond archipelago in southeastern France. We investigated the interactions between vital rates (survival and recruitment), the internal structure of the population, and climatic variables both at a local and a regional (North Atlantic Oscillation: NAO) scale. Overall, we found a weak relationship between climatic variables and the survival of large-bodied newts. The only strong relationship was found to be a high NAO index during the post-breeding period, suggesting that dry, hot summers negatively impact survival. In terms of recruitment, the results indicated that hot weather during the activity period had delayed deleterious effects on adult recruitment two years later, suggesting high larval and juvenile mortality due to unsuitable growing conditions. Recruitment was also impacted by a high NAO index during the overwintering period preceding recruitment, suggesting that mild weather increases the mortality of juveniles, probably by enhancing the depletion of energy reserves without any possibility of refueling.

## Introduction

There is no longer any doubt that the climate is changing on a global scale, as mean surface temperatures have increased by approximately 0.2 °C per decade over the past 30 years^1^. Water availability is also changing, with global precipitation increasing overall but exhibiting high temporal and spatial disparities^2^. Extreme weather events, such as heavy rainfall or severe drought, are reported to be increasing in both frequency and intensity^3^. These environmental changes are expected to severely impact biodiversity, leading to range shifts, long-term population decline, and, ultimately, the extinction of many species^4^.

During the last three decades, many studies have shown that weather variations have an impact on population dynamics through complex effects on survival and recruitment^4^. Weather variation can affect an individual’s physiological state directly, resulting in increased mortality risks, especially during energy-demanding activities such as growth and reproduction^5^. Weather fluctuations can also indirectly impact survival by affecting both the availability and the quality of feeding resources^6,7^ or by enhancing predation risks^8^. Recruitment may also be influenced by weather fluctuations. First, this can be due to a trade-off between the expected fitness benefits of the current breeding cycle and those of future reproduction^9,10^. Weather fluctuations can influence a female’s breeding decisions^11^ as well as investment in breeding^12^, which can further affect fecundity and recruitment. Second, by affecting fat storage, oogenesis and gestation in females, weather variations can indirectly impact the body condition, development and survival of offspring after hatching or parturition through maternal effects^13^. Third, by influencing both the abiotic (e.g. temperature, amount of water) and biotic (e.g. food availability, predator density) conditions that prevail during the juvenile stage, weather conditions can impact growth and survival and thus future reproductive performance^14–16^, which can then lead to strong variations in recruitment.

Although many studies have investigated the demographic responses to weather variations in endotherms (review in^4^), knowledge remains fragmentary in terrestrial ectotherms despite the fact that the population dynamics of these organisms are expected to be highly sensitive to weather fluctuations^17^. Amphibians are often considered valuable candidates for such investigations, since temperature, rainfall and snowpack are assumed to have a strong influence on their vital rates^18^. A recent analysis based on long-term monitoring (>10 years) of 31 populations belonging to 11 species distributed over three climatic zones in Europe and North America has shown the great complexity of the relationship between climatic variables and vital rates^19^. The results indicated that climate influences vital rates, but with great variation between taxa and between populations within taxa, suggesting a context-dependent response to climatic variation. While winter-related covariates were most frequently correlated to vital rates, the direction of the relationship varied between taxa and between populations. Warm weather conditions during winter may result in depletion of energy reserves while the animal is unable to feed, which may in turn affect breeding decisions or impair offspring development due to maternal effects^20–22^. However, some studies have highlighted an opposite effect with low temperatures affecting survival^23,24^. Droughts have a negative impact on adult survival^6,25^, probably due to the deleterious effects of dehydration in these animals, which have no protection against transcutaneous water loss^26^. Rainfall deficits can also negatively influence female breeding probability and investment^6,27^. Finally, as weather variations strongly influence local hydrological regimes, they can negatively affect larval and juvenile survival. For instance, excess rainfall resulting in extreme flooding can negatively affect juvenile survival^27^, while a rainfall deficit can lead to ponds drying out, causing catastrophic larval mortality^28^.

Despite these valuable efforts to capture general trends about the relationships between climate and demography, the level of predictability remains low and is strongly context dependent. In light of this, in this study we investigated climatic interactions with variables linked to the internal process of population functioning. At the intra-population level, little is known about how interactions between weather and internal factors, such as body size, sexual maturity and gender, may affect demographic parameters^20,29^. This point has been neglected, while it seems crucial in understanding amphibian demographic responses to weather fluctuations in the context of climate change.

To fill this gap, we investigated the relationships between weather variation and key demographic parameters in a great crested newt (*Triturus cristatus*) population that had been monitored for over 20 years. We chose this species because of its large body size, which allows the use of Passive Integrated Transponder (PIT) tags, and its low population density, allowing the optimization of capture costs. It can be considered a suitable sentinel species for investigating the interactions of population dynamics and environmental variation. Using long-term capture–recapture data, we examined the relationships between weather conditions and both survival and recruitment, taking into account body size, sexual maturity and gender. We related local weather variables (i.e. temperature, precipitation and number of frost and snow days) and regional weather patterns (North Atlantic Oscillations) to survival during three periods: the breeding period (from March to May), the post-breeding (or active) period (June to October), and the overwintering (or resting) period (November to February). North Atlantic Oscillations (NAO) derive from differences in atmospheric pressure between the Azores and Iceland. As this index reflects large-scale weather variations, it can be considered as an integrator of different metrics. High NAO values are related to mild, wet winters in northern Europe and warm, dry winters in southern Europe^30,31^. Based on recent studies, we made hypotheses about specific survival responses during the newt’s biological cycle depending on weather conditions: we hypothesized that survival should be impacted by water deficits during the active period and by warm weather during the resting period (Table 1). We also related weather factors to local recruitment of adult individuals (i.e. the proportion of breeders recruited each year to the population). We expected that both local weather variables (temperature and precipitation) and the NAO would influence the recruitment of new adults through their impact on larval development (on average two years before recruitment, as an individual reaches sexual maturity at the age of three (ref.^32^ and unpubl. results), and juvenile growth (on average one year before recruitment). We expected water deficits to be negatively related to recruitment two years later, and both low temperature and/or water deficits to be negatively related to recruitment one year later. We also expected juveniles to be more sensitive to weather conditions because (i) they have a higher ratio of skin surface to body mass and (ii) they may exhibit more risk-prone behavior as they need to forage intensively in order to reach sexual maturity^33^. We also examined whether recruitment could be influenced by weather variations during winter, one or two years before recruitment, due to the deleterious impact of mild weather on the newts’ energy reserves^19,21^.

**Table 1:** Hypotheses regarding the potential impact of selected weather factors on demographic parameters.

	Recruitment			Adult survival		
	Larval development	Juvenile active period	Juvenile overwintering	Breeding period	Active period	Overwintering
Weather factor	Temperature^a,c^ (−)	Temperature (−)	Snow^e,f^ (+)	Temperature^b,d^ (−)	Temperature (−)	Snow^c,e^ (+)
	Rainfall^a,c^ (+)	Rainfall (+)	Frost^e,f^ (+)	Rainfall^b,d^ (+)	Rainfall (+)	Frost^c,e^ (+)
	NAO^a,c^ (−)	NAO (−)	NAO^e,f^ (−)	NAO^b,d^ (−)	NAO (−)	NAO^c,e^ (−)

The sign (+ or −) gives the expected effect direction of the factor on each parameter. These exponents refer to publications where the relationship has been investigated (a^62^, b^63^, c^29^, d^64^, e^21^, f^20^)

## Materials and Methods

### Study area, field sampling and weather data

Water-breeding amphibians often breed in pond archipelagos, so populations have a polynodal spatial structure. To take this into account, the sampling data was collected in an archipelago of 12 ponds created for monitoring purposes in the private nature reserve of the Pierre Vérots Foundation on the Dombes Plateau in France (45°56′N, 4°55′E). Five ponds were constructed in 1992, two others in 1996, and five more in 1999. Wild dispersing newts spontaneously colonized the ponds – they were not introduced. Many ponds are scattered over the study region, explaining the presence of the species in the close surroundings. The distance between ponds varied from 30 m to 430 m. Pond morphology was standardized to a surface area of 50 m^2^ (5 × 10 m) with a depth of 1.5 m at the deepest point. Two plant species, *Juncus effusus* and *Glyceria sp*., quickly colonized all the ponds, with *Juncus* occupying the banks and *Glyceria* the zones with a depth between 30 and 70 cm. The deepest zone was not occupied by any plants. Other plants were occasionally present (*Ranunculus aquaticus, Potamogeton natans, Callitriche* sp*., Alisma plantago*), but their presence was stochastic. It is likely that the use of a seine net to fish out newts during the monitoring process caused a disturbance effect that contributed to the stability of the vegetation pattern. As the ponds were located within a protected nature reserve, the surrounding landscape remained unchanged throughout the monitoring period.

Sampling of the ponds was performed from 1996 to 2015. Two capture sessions were carried out per year during the breeding season, when the newts live underwater: the first was carried out in late March and the second in late April. The newts were caught using a fishing net (a 2 × 10 m seine) repeatedly pulled through the water until no more were caught in the net, after a minimum of three net passages per pond. Each individual was marked with a PIT tag (Biolog-Id, France), a technique known to have little impact on survival and body condition^34^. At each capture, the stage (juvenile or adult), gender (in adults), body size (snout–vent length in mm, 0.5 mm accuracy) and mass (in g, 0.1 g accuracy) were recorded. The gender was determined based on the presence of a swollen cloaca and a large crest on the back in males^35^. Body size was then categorized into three groups: small (40–55 mm), intermediate (55–70 mm), and large (above 70 mm), including large juveniles (sufficiently large to be marked, i.e. above 3 g).

Local weather conditions were noted using standard meteorological indices provided by the Meteo France database (publitheque.meteo.fr). The selected metrics (mean daily temperature [°C] and rainfall [mm]) were recorded at the Marlieux meteorological station (46°02′N, 5°02′E), located 20 km from the study site on the same geological plateau, and they were computed to obtain monthly averages. For the overwintering period, we also included the number of frost days and the number of days with lying snow as indicators of weather conditions. Additionally, as repeated collinearity between environmental predictors usually prevents the inclusion of multiple weather variables in models, we also opted to use the NAO index as an integrator variable. This can be correlated to local indices to get an idea of the significance of NAO variations on a local scale^36^. The NAO values were provided by the open-access database from the Climatic Research Unit, University of East Anglia (crudata.uea.ac.uk/cru/data/nao).

### Hypotheses concerning recapture probability

Before building capture–recapture models based on the data from the long-term monitoring study, we performed a goodness-of-fit test using the U-CARE program^37^. We tested both the full dataset and a subset containing only adults, as juveniles were included only in the analysis of survival (not of recruitment). The TEST3.SR analysis for potential transience per year^38,39^ showed a significant number of transients in the population if juveniles were taken into account in the analysis (df = 18, χ^2^ = 72.438, *P* < 0.001). This analysis found that 6 out of the 18 sampling years exhibited a significant number of transients (*P* < 0.05). If the capture histories of adult individuals only were considered, there was not a significant number of transients (df = 18, χ^2^ = 25.169, *P* = 0.12) across the whole series. Transience in adults reached a significant level only during two years (*P* < 0.05). As most of the transience was due to the permanent emigration of juvenile newts, we needed to work with demographic models with explicit maturity status. Given that certain years had a high level of transience, we specifically developed a capture–recapture multi-event model that allows segregating transient and resident individuals. It should be noted that this model might slightly overestimate the survival rate of juveniles, since a proportion of dead newts may be measured as transients.

### Modeling survival

We first investigated the influences of body size, gender, maturity status and weather factors on survival. To deal with sex uncertainty in juveniles and the high level of transience, we used multi-event models^40^, including initial state probabilities, matrices of state–state transition probabilities, and the matrix of observation probabilities linking the events (i.e. field observations) and the underlying ‘true’ states of the individuals (Supplementary File SF1, Events). We considered three states according to size, maturity and gender, resident/transient individuals. This led to the consideration of 21 states and 9 events (SF6.1 and 6.2). To designing initial state probabilities, we first estimated the probability that an individual was transient or resident, and second we attributed non-transient individuals to one of the nine states and transient individuals to one of the ten possible states (SF1: Initial states).

From these initial states, in the first stage of the state–state transition process, the survival status of each individual was updated (STEP1 in SF1: Survival). Note that the survival of transients is by definition forced to 0. In a second stage, the individuals that survived were allowed to change size group (STEP2 in SF1). In the third stage, the maturity status was updated (STEP3 in SF1). Finally, events were linked to the true underlying states of the individuals, and individuals could be recaptured with probability *p* or not captured (NC) with probability 1–*p* (SF1: Events).

This parameterization was implemented with E-SURGE^41^ using the Akaike information criterion corrected for small sample size (AICc)^42^. The analyses were carried out in two steps. First, from the most general model, *δ*(*T*)*Φ*(*SVL* × *SEX* + *T*)*p*(*SVL* × *SEX* + *T*), we examined through a backward stepwise selection whether recapture, then transience, and, lastly, survival depended on the three following covariates: body size, gender and year-specific variations. As gender assessment relied on the presence of secondary sexual characteristics, this could only be ascertained for mature individuals, leading to the need for three modalities: juveniles, males and females. Adult abundance was derived from the best-fit model. In a second step, after determining the best model with internal state variables, we examined the relationships between weather covariates and survival using ANODEV, as recommended by Grosbois *et al*.^5^. Each weather covariate was tested in turn, and its explanatory power quantified with the analysis of deviance. This approach allowed us to evaluate the fit of a model including a single weather covariate (*M*_*co*_) involving both constant (*M*_*cst*_) and time-dependent (*M*_*t*_) models. The statistical *Ftest*_*cst*/*co*/*t*_ was derived as follows:

$$Ftes{t}_{cst/co/t}=(\frac{Dev({M}_{cst})-Dev({M}_{co})}{\zeta -1})$$

This procedure tests the null hypothesis *H*_*o*_ that the weather covariate in *M*_*co*_ has no significant relation with survival. Under *H*_*o*_, it follows a Fisher-Snedecor distribution with *ζ* − 1 and n − *ζ* degrees of freedom, where *n* is the number of demographic parameter estimates and *ζ* is the number of parameters required to describe the relationship. We tested the effect of the weather covariates on the three stages of a newt’s biological cycle (Table 1).

### Modeling recruitment

Because we were only interested in adult recruitment, capture–recapture histories of juveniles were eliminated from the dataset. We extended Pradel’s seniority model^43^ to estimate recruitment while including transience; the model calculates the probability of not being in the system the year before, i.e. of being ‘recruited’ during the current year. For this purpose, we reversed the capture histories in order to use the survival models^41^ in the multi-event context. This led to the consideration of three states (transient, resident, and not yet recruited) and two events (not captured and captured). As with the survival model, the transient/resident state was updated in the initial state of departure step (SF2: Transience). The recruitment probability was then updated (SF2: Recruitment). Concerning events, an individual could be recaptured with probability *p*, or not recaptured with probability 1–*p* (SF2: Events).

This parameterization was implemented with E-SURGE software^39^. Analyses were carried out following the same procedure used for survival. First, through a backward stepwise selection from the most general model, *δ*(*SEX* + *T*) *r*(*SEX* + *T*) *p*(*SEX* + *T*), we tested the hypotheses that adult transience, recruitment and recapture could vary according to gender and between years. We first fixed the structure of the recapture component of the model, then the transience component, and finally tested hypotheses on recruitment. Once the structure of the best-fit model was ascertained, the relationship of each weather covariate with recruitment was tested individually, and its explanatory power quantified with ANODEV.

As crested newts usually reach sexual maturity at the age of 2 (or more rarely 3) in the populations of the study area (ref.^32^ and unpubl. res.), we examined correlations between recruitment and weather factors recorded either one or two years before adult recruitment. We considered the weather variables recorded during three periods: (i) at the time of larval development two years before recruitment, (ii) at the time of juvenile development (active period) one year before recruitment, and (iii) at the time of overwintering two years and one year before recruitment (Table 1). During breeding activity, we examined whether recruitment depended on: (1) mean temperature during larval development two years before recruitment, (2) during juvenile development (active period) one year before recruitment, and (3) cumulative rainfall two years and (4) one year before recruitment. During the winter period we tested the same metrics as in the survival model, but recorded one or two years before maturity, thus corresponding to first and second juvenile overwintering. We also tested the correlation with the NAO index for all the aforementioned periods. The structure of modeling, the results of model selection and the meaning of the abbreviations are given in the supplementary files.

### Ethical rules

The crested newt is a protected species in France and in Europe. This study was authorized by the Prefect of the Ain department (Derogation No. DDPP01-16-190), and the Ethical Committee of Lyon University approved all the experiments that were carried out. PJ and OG were given approval for performing experiments with living vertebrates, including transponder injection. They strictly followed all the relevant guidelines and regulations for the use of animals in experimental research.

## Results

### Abundance

**Figure 1:**
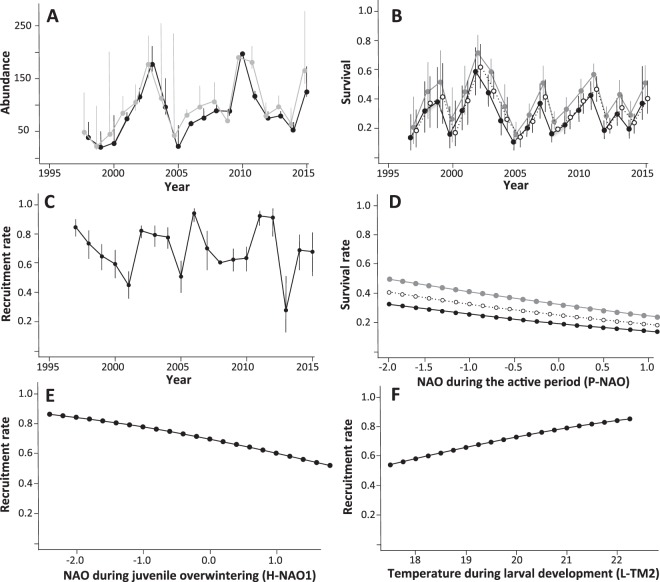
Variation of demographic parameters over time (1996–2015) (**A–C**), and relationships between them and weather variables (**D–F**), in a crested newt population (*Triturus cristatus*) monitored by mark & recapture in southeastern France. (**A**) Variation in adult abundance (grey = females; black = males); (**B**) Variation in survival rate (open dots = small-sized [40–55 mm]; grey dots = intermediate [55–70 mm]; black dots = large [<70 mm]). (**C**) Variation in recruitment rate. (**D**) Relationship between NAO during the active period (P-NAO) and survival rate (open = small; grey = intermediate; black = large). (**E**) Relationship between NAO during juvenile overwintering (H-NAO1) and recruitment rate. (**F**) Relationship between temperature during larval development (L-MT2) and recruitment rate. Error bars correspond to the confidence intervals.

Throughout the monitoring period, 3,745 individuals (1,173 males, 1,237 females and 1,335 juveniles) were captured. The number of individuals captured varied greatly from year to year, with a minimum of 60 in 1999 to a maximum of 516 in 2010. The abundance of adult newts also fluctuated widely between years (Fig. 1A). After a continuous increase in abundance following colonization of the ponds, a first drop occurred in 2003–04, reducing the population to its level during the first years of monitoring. After this drop, abundance rose again until another drop occurred in 2012–13. These two population declines were associated with two severe drought/heatwave events (2003 and 2011), identified as exceptional by the French weather service (MétéoFrance).

### Survival analysis

In the first step of the survival analysis, without weather covariates, the best-fit models were *δ*(*T*)*Φ*(*SVL* × *SEX* + *T*), *p*(*SEX* + *T*) and *δ*(*T*), *Φ*(*SVL* + *T*)*p*(*SEX* + *T*), with *δ* for transience variation, *Φ* for survival and *p* for capture probability, depending on time (T), size (SVL), and gender (SEX). Their QAICc differed by less than one point, making it impossible to discriminate between them (Anderson and Burnham, 2002). Following the parsimony principle, we opted to retain the simplest for testing the effects of climate on survival (SF7, models 1 and 2). In this model, the transience rate did not depend on body size, gender or maturity, but varied between years: from 0 in 2005 to 0.54 (95% confidence interval [CI] 0.42–0.66) in 2004. Survival probability varied according to body size, and also fluctuated between years (Fig. 1B). The mean survival rate (extracted from the constant model) was 0.35 (95% CI 0.33–0.37). Mean survival was higher in individuals with an intermediate body size (0.39 [95% CI 0.36–0.42]), and lower in both small (0.31 [95% CI 0.28–0.34]) and large (0.26 [95% CI 0.22–0.32]) individuals (Fig. 1B). Survival was also highly variable between years: in small individuals survival ranged from 0.1 (95% CI 0.05–0.17) in 2005 to 0.62 (95% CI 0.45–0.75) in 2002. In intermediate-sized individuals, survival varied from 0.14 (95% CI 0.08–0.22) in 2005 to 0.7 (95% CI 0.55–0.81) in 2002. In large individuals, survival ranged from 0.08 (95% CI 0.05–0.15] in 2005 to 0.57 (95% CI 0.41–0.73) in 2002.

Recapture probability also fluctuated depending on status, with greater recapture rates for males (0.92 [95% CI 0.86–0.96]) and females (0.86 [95% CI 0.80–0.91]) than for juveniles (0.48 [95% CI 0.27–0.70]). Recapture probability fluctuated depending on time ranging from 0.12 (95% CI 0.07–0.2) in 2005 to 0.69 (95% CI 0.52–0.82) in 2002. The AICc of the best models with weather covariates revealed that most fit much better than the constant model, but much less than the time-varying model (SF7 and 8). The QAICc of the best-fit models with weather covariates was 25 points (for P-NAO) lower than that of the constant model, showing a significant difference from the constant model^44^. Our results thus showed a decrease in survival when the NAO index increased during the post-reproductive period (slope coefficient: −0.25 [95% CI −0.35–−0.15], Fig. 1D). However, the ANODEV showed that this gain in AICc points was minimal and that the majority of the variation in yearly survival remained unexplained (F = 3.27, *P* = 0.09).

### Recruitment analysis

In the recruitment analysis, the best-fit model without weather covariates was δ(.), r(T), p(Sex), with δ for transience, *r* for recruited, and *p* for capture probability, depending on time (T) and gender (SEX) (SF9). The transience rate did not differ between genders and was constant between years (0.10 [95% CI 0.03–0.30]). The recruitment rate varied strongly between years (Fig. 1C), ranging from 0.27 [95% CI 0.12–0.5] in 2013 to 0.94 [95% CI 0.88–0.97] in 2006. The mean recruitment rate was relatively high (0.71 ± 0.02). The recapture rate differed between genders, with males (0.81 [95% CI 0.73–0.87]) more frequently recaptured than females (0.62 [95% CI 0.52–0.70]).

ANODEV showed that two models that included weather covariates explained a significant part of the temporal variation in recruitment (SF10): the model incorporating winter NAO one year before maturity (H-NAO1, F = 15.72, *P* = 0.001), and the model including the mean temperature during larval development two years before maturity (L-MT2, F = 7.47, *P* = 0.014). Our results showed that recruitment was negatively related to H-NAO during juvenile development (one year before recruitment) (slope coefficient: −0.54 [95% CI −0.64–−0.44], Fig. 1E), suggesting a deleterious effect of mild weather on juvenile survival during winter. In contrast, it was positively correlated to mean temperature during larval development (two years before recruitment) (slope coefficient: 0.49 [95% CI 0.37–0.61], Fig. 1F), suggesting that high temperatures promote survival during the larval stage.

## Discussion

As declines in abundance occurred after each of the two main drought events recorded during the monitoring period (in 2003 and 2011), we expected to detect strong relationships between demographic parameters and climatic variables. In fact, we found that survival was only marginally related to weather variation, although recruitment proved to be more sensitive to this, as it was positively related to mean temperature during larval growth and negatively related to winter NAO during juvenile overwintering.

Adult survival (ranging from 0.26 to 0.39 depending on body size) was lower than in other populations of the same species surveyed in Western Europe (around 0.50 in^21^ and^45^). This lower survival rate may have resulted from substantial permanent emigration from the ponds studied, as our models (as is usual in CR analyses) estimated apparent survival (local survival), which typically includes both death and permanent emigration^46^. The high density of the ponds and their convenient layout providing easy access for newts are features that might enhance the emigration rate^47^. Nonetheless, the fact that skeletochronological studies of this population suggest a lower rate of aging (the oldest recorded age of an individual was 11 years, whereas a study in a neighboring region recorded individuals of 18 years^43^), suggests a lower mean survival rate in our population.

Our analyses also revealed that survival differed according to body size. Survival was lowest (0.26) in small individuals (40–55 mm), which may have two non-mutually exclusive explanations. First, as natal dispersal is usually thought to be the main mechanism of dispersal in crested newts^21^, one might expect survival to be biased by permanent emigration from the study area. Second, lower survival in small individuals than in large individuals might result from exposure to a wider range of predators, a less efficient immune system, lack of experience and/or an energy allocation pattern that favors growth over maintenance, thus leading to individuals displaying risk-prone foraging activities. Such a lower survival in juveniles has been observed in other amphibian species in which survival is positively related to body size after metamorphosis^48,49^. In our population, survival was highest (0.39) in individuals with an intermediate body size (from 55 to 70 mm) and decreased in large individuals (0.31). As locomotive performance is positively related to body size in urodeles^49^, this could result in increased dispersal in large individuals. However, other studies on the relationship between body size and dispersal in our study population (not yet published) do not support this hypothesis. Another reason for this decrease in survival in the largest newts could be actuarial senescence (i.e. the increase in mortality with age). This is difficult to prove since senescence mechanisms remain poorly understood in amphibians^50^ and in ectothermic vertebrates in general. Further studies are needed to better understand the survival pattern we found in the present study.

Regarding the impact of weather, despite high temporal variations, our analyses failed to detect substantial effects of single weather factors, such as temperature and precipitation, on survival. Rather, we detected a marginal negative relationship between the NAO index and survival during the post-breeding period. As high NAO index values correspond to dry, warm summers in the study area (see SF1), this suggests that drought has a slight negative impact on survival at the end of the active period. Such a trend is congruent with other studies that have reported deleterious effects of drought on survival in other amphibians (e.g.)^51^. This result suggests that a water deficit may impact survival during the post-nuptial activity period (which involves landward migration and foraging activities on land), thus contributing to the two declines in abundance we observed. However, climatic variables only seemed to have a relatively slight impact on survival variation, so other factors have to be considered. These may include positive density-dependence after a population decline or during the colonization of ponds experiencing the early stages of ecological succession.

Our results diverge from those of Griffiths *et al*.^21^, who found a significant negative effect of heavy rainfall and mild temperatures during the overwintering period on crested newt population dynamics. According to these authors, mild weather during overwintering could enhance metabolism, leading to wasting of energy reserves, and heavy rainfall could disturb gas exchanges, as in the case of water-saturated soil, both effects resulting in decreased survival. The fact that we did not detect similar relationships could be due to regional differences in soil structure, rainfall pattern and the type of winter refuge used by the newts. Despite limited knowledge of the ecology of overwintering newts, we suspect that they mainly use rodent burrows as shelters. In a radio-tracking study that monitored post-breeding migrations of the Italian crested newt (*T. carnifex*, closely related to the great crested newt), Schabetsberger *et al*.^52^ found most newts in rodent burrows at depths varying from 5 to 80 cm. Such variation in depth of a newt’s winter refuge is likely to lead to great variations in the abiotic conditions experienced during the resting period. The architecture of rodent burrows is strongly influenced by both local soil and vegetation structure^53,54^, thus creating subterranean conditions that are likely very dependent on local conditions. Furthermore, the intensity of both rainfall and temperature variation may differ between the population monitored by R. Griffiths in southern England and our population in the Rhône-Alpes region.

We found that recruitment varied greatly between years, and this variation was partly explained by weather effects. In our study, recruitment was defined as the proportion of adults recruited to the population each year; this includes both adult immigration and local reproduction output. Whatever the process, both locally produced and immigrant newts are assumed to have experienced similar weather conditions, thus allowing recruitment to be considered as a homogeneous variable. We detected substantial delayed effects of weather variations on recruitment rates. The recruitment rate was positively related to the mean temperature of the active period two years before: i.e. during the larval stage for most of the recruited individuals. Except in the case of excessive temperatures leading to pond drying and massive larval mortality, high temperature is expected to stimulate productivity, leading to high resource availability, high growth rates and large size at metamorphosis^55^. However, several studies have shown that high growth rate can result in a smaller size at metamorphosis^18,56^, leading to a lower survival rate during the first overwintering, especially in populations at high elevations^44^.

Our results also showed that recruitment was negatively related to a high NAO index during the winter preceding adult recruitment. In our study area, a high NAO index corresponds to relatively warm and wet conditions during newt overwintering (SF2 for the correlation matrices). In contrast with what we observed with large newts (sufficiently large to be tagged), this result converged with previous studies reporting that mild winters usually result in hibernating amphibians continuing to deplete energy reserves while unable to feed, leading to a low survival rate the following spring^20,21^. This discrepancy in survival rates between large and small (i.e. juvenile) newts might be explained by the fact that the specific energy demands of juvenile newts could be higher than those of adults due to an allometric relationship between body mass and metabolic rate^57,58^. Furthermore, juvenile newts are expected to exhibit more risk-prone behavior with respect to overwintering conditions because of the positive consequences on fitness of high growth rates before sexual maturity is reached.

The relationships we found between climatic variables and demographic parameters at least partly explain the impact of severe droughts on abundance. However, analyzing the consequences of catastrophic events within a long-term monitoring framework remains a challenge^21,59,60^, since the relationship between weather and demographic parameters during regular years is likely to underestimate their importance^61^. That being said, it remains true that the impact of weather variation on the dynamics of our crested newt population was relatively weak. This conclusion diverges from the mainstream opinion according to which the amphibians are valuable biological indicators of climate change because their water balance tightly depends on environmental conditions. Our population indeed exhibits a good resilience to weather variation, the stability of adult survival buffering variation in larval and juvenile survival induced by droughts or mild winters. Probably the adults benefit from a low skin area on mass ratio, and a low investment in growth, and finally a behavioural control of homeostasis through shelter selection that affords them an efficient guarantee against harsh weather conditions. However, the resilience of this population cannot be extrapolated to other populations of the same species at other localities, nor to populations of other species, because of the complexity of the interplay between local weather patterns, population pace of life and local ecological background^19^.

In addition, despite the relatively long duration of the monitoring at the scale of the professional lifespan of a researcher, it may be questioned if this duration is sufficient for predicting the impact of climate change on newt populations. In the population under study, twenty years is the time needed for the turnover of only 4- or 5-generations and for recording the effects of two main drought events, which could be considered a limited number for a comprehensive understanding of the operating demographic processes. Furthermore, this monitoring duration is also too short for gaining a predictive insight about the way by which climate change will be expressed at a local scale. If the frequency of drought events or of mild winters increases, thus increasing larval and juvenile mortality, it becomes possible that the compensatory effects of adult survival will not be sufficient to sustain population dynamics.

Considering the complexity and the diversity of the relationships between weather covariates and vital rates^19^, our findings highlight the value of exploring the relationships of these covariates with internal population processes. This study is a first step in this direction; further studies that integrate other internal processes such as density-dependence, dispersal, and temporal auto-correlation will bring complementary insights. The ultimate goal would be to build a comprehensive model that integrates species-specific demographic process (including rules of density-dependence), weather variation and consecutive changes in the dynamics of vegetation and prey productivity.

## Electronic supplementary material

Supplementary Information

## Data Availability

The data used in this publication will be available in the Zenodo database.
