# Supplementary material for: Resilience to climate variation in a spatially structured amphibian population
Source: Sci Rep. 2018 Oct 2;8:14607. doi: 10.1038/s41598-018-33111-9 (PMC6168496; doi:10.1038/s41598-018-33111-9)
Supplement: Supplementary file 1 — Supplementary Information [file 41598_2018_33111_MOESM1_ESM.pdf]

Resilience to climate variation in a spatially structured amphibian population

A. Weinbach, H. Cayuela, O. Grolet, A. Besnard and P. Joly

Supplementary files

S.F. 1. Correlation between the NAO and local weather descriptors. Definitions of the abbreviations in S.F. 5.

	R-NAO	R-MT	R-RD
R-NAO	-	0.61	-0.34
R-MT	-	-	-0.39
R-RD	-	-	-

	P-NAO	P-MT	P-RD
P-NAO	-	0.04	-0.49
P-MT	-	-	-0.15
R-RD	-	-	-

	H-NAO	H-SNOW	H-FROST
H-NAO	-	-0.66	-0.39
H-SNOW	-	-	0.63
H-FROST	-	-	-

	L-NAO1	L-MT1	L-RD1
L-NAO1	-	-0.04	-0.21
L-MT1	-	-	0.33
L-RD1	-	-	-

	L-NAO2	L-MT2	L-RD2
L-NAO2	-	-0.09	-0.43
L-MT2	-	-	-0.33
L-RD2	-	-	-

	H-NAO1	H-SNOW1	H-FROST1
H-NAO1	-	-0.66	-0.34
H-SNOW1	-	-	0.57
H-FROST1	-	-	-

	H-NAO2	H-SNOW2	H-FROST2
H-NAO2	-	-0.60	-0.40
H-SNOW2	-	-	0.49
H-FROST2	-	-	-

S.F. 2. Matrices of the survival model. †: dead. For a comprehensive list of the other state abbreviations (e.g. RSMi) see S.F. 6.

Transience

R	T
$1-\delta$	δ

Initial states

	RSMi	RSMm	RIMi	RIMm	RLMm	RSFi	RSFm	RIFi	RIFm	RLFm	TSMi	TSMm	TIMi	TIMm	TLMm	TSFi	TSFm	TIFi	TIFm	TLFM
R	η	η	η	η	η	η	η	η	$1-\eta$	0	0	0	0	0	0	0	0	0	0	0
T	0	0	0	0	0	0	0	0	0	η	η	η	η	η	η	η	η	η	η	$1-\eta$

STEP2: Length

	RSMi	RSMm	RIMi	RIMm	RLMm	RSFi	RSFm	RIFi	RIFm	RLFm	†
RSMi	ε	0	$1-\varepsilon$	0	0	0	0	0	0	0	0
RSMm	0	ε	0	$1-\varepsilon$	0	0	0	0	0	0	0
RIMi	0	0	1	0	0	0	0	0	0	0	0
RIMm	0	0	0	ε	$1-\varepsilon$	0	0	0	0	0	0
RLMm	0	0	0	0	1	0	0	0	0	0	0
RSFi	0	0	0	0	0	ε	0	$1-\varepsilon$	0	0	0
RSFm	0	0	0	0	0	0	ε	0	$1-\varepsilon$	0	0
RIFi	0	0	0	0	0	0	0	1	0	0	0
RIFm	0	0	0	0	0	0	0	0	ε	$1-\varepsilon$	0
RLFm	0	0	0	0	0	0	0	0	0	1	0
†	0	0	0	0	0	0	0	0	0	0	1

STEP1: Survival

	RSMi	RSMm	RIMi	RIMm	RLMm	RSFi	RSFm	RIFi	RIFm	RLFm	†
RSMi	ϕ	0	0	0	0	0	0	0	0	0	$1-\phi$
RSMm	0	ϕ	0	0	0	0	0	0	0	0	$1-\phi$
RIMi	0	0	ϕ	0	0	0	0	0	0	0	$1-\phi$
RIMm	0	0	0	ϕ	0	0	0	0	0	0	$1-\phi$
RLMm	0	0	0	0	ϕ	0	0	0	0	0	$1-\phi$
RSFi	0	0	0	0	0	ϕ	0	0	0	0	$1-\phi$
RSFm	0	0	0	0	0	0	ϕ	0	0	0	$1-\phi$
RIFi	0	0	0	0	0	0	0	ϕ	0	0	$1-\phi$
RIFm	0	0	0	0	0	0	0	0	ϕ	0	$1-\phi$
RLFm	0	0	0	0	0	0	0	0	0	ϕ	$1-\phi$
TSMi	0	0	0	0	0	0	0	0	0	0	1
TSMm	0	0	0	0	0	0	0	0	0	0	1
TIMi	0	0	0	0	0	0	0	0	0	0	1
TIMm	0	0	0	0	0	0	0	0	0	0	1
TLMm	0	0	0	0	0	0	0	0	0	0	1
TSFi	0	0	0	0	0	0	0	0	0	0	1
TSFm	0	0	0	0	0	0	0	0	0	0	1
TIFi	0	0	0	0	0	0	0	0	0	0	1
TIFm	0	0	0	0	0	0	0	0	0	0	1
TLFM	0	0	0	0	0	0	0	0	0	0	1
†	0	0	0	0	0	0	0	0	0	0	1

Events

	NC	Si	SMm	Ii	IMm	LMm	SFm	IFm	LFm
RSMi	$1-p$	p	0	0	0	0	0	0	0
RSMm	$1-p$	0	p	0	0	0	0	0	0
RIMi	$1-p$	0	0	p	0	0	0	0	0
RIMm	$1-p$	0	0	0	p	0	0	0	0
RLMm	$1-p$	0	0	0	0	p	0	0	0
RSFi	$1-p$	p	0	0	0	0	0	0	0
RSFm	$1-p$	0	0	0	0	0	p	0	0
RIFi	$1-p$	0	0	p	0	0	0	0	0
RIFm	$1-p$	0	0	0	0	0	0	p	0
RLFm	$1-p$	0	0	0	0	0	0	0	p
TSMi	$1-p$	p	0	0	0	0	0	0	0
TSMm	$1-p$	0	p	0	0	0	0	0	0
TIMi	$1-p$	0	0	p	0	0	0	0	0
TIMm	$1-p$	0	0	0	p	0	0	0	0
TLMm	$1-p$	0	0	0	0	p	0	0	0
TSFi	$1-p$	p	0	0	0	0	0	0	0
TSFm	$1-p$	0	0	0	0	0	p	0	0
TIFi	$1-p$	0	0	p	0	0	0	0	0
TIFm	$1-p$	0	0	0	0	0	0	p	0
TLFM	$1-p$	0	0	0	0	0	0	0	p
†	1	0	0	0	0	0	0	0	0

STEP3: Maturity

	RSMi	RSMm	RIMi	RIMm	RLMm	RSFi	RSFm	RIFi	RIFm	RLFm	TSMi	TSMm	TIMi	TIMm	TLMm	TSFi	TSFm	TIFi	TIFm	TLFM	†
RSMi	$1-\gamma$	γ	0	0	0	0	0	0	0	0	0	0	0	0	0	0	0	0	0	0	0
RSMm	0	1	0	0	0	0	0	0	0	0	0	0	0	0	0	0	0	0	0	0	0
RIMi	0	0	$1-\gamma$	γ	0	0	0	0	0	0	0	0	0	0	0	0	0	0	0	0	0
RIMm	0	0	0	1	0	0	0	0	0	0	0	0	0	0	0	0	0	0	0	0	0
RLMm	0	0	0	0	1	0	0	0	0	0	0	0	0	0	0	0	0	0	0	0	0
RSFi	0	0	0	0	0	$1-\gamma$	γ	0	0	0	0	0	0	0	0	0	0	0	0	0	0
RSFm	0	0	0	0	0	0	1	0	0	0	0	0	0	0	0	0	0	0	0	0	0
RIFi	0	0	0	0	0	0	0	$1-\gamma$	γ	0	0	0	0	0	0	0	0	0	0	0	0
RIFm	0	0	0	0	0	0	0	0	1	0	0	0	0	0	0	0	0	0	0	0	0
RLFm	0	0	0	0	0	0	0	0	0	1	0	0	0	0	0	0	0	0	0	0	0
†	0	0	0	0	0	0	0	0	0	0	0	0	0	0	0	0	0	0	0	0	1

S.F. 3: Matrices of the recruitment model. R: resident, T: transient, NC: not captured, C: captured, †: not yet recruited.

Transience

$$\begin{array}{cc} R & T \\ \end{array}$$
$$\begin{pmatrix} 1-\delta & \delta \end{pmatrix}$$

Recruitment

$$\begin{array}{c|ccc} & R & T & \dagger \\ \hline R & 1-r & 0 & r \\ T & 0 & 0 & 1 \\ \dagger & 0 & 0 & 1 \end{array}$$

Events

$$\begin{array}{c|cc} & NC & C \\ \hline R & 1-p & p \\ T & 1-p & p \\ \dagger & 1 & 0 \end{array}$$

S.F. 4: Variations of the different weather indicators

	min	max	mean	med	var
R-MTa	9.87	13.83	11.73	11.63	0.94
R-RD	88.50	374.70	210.05	207.00	5372.88
R-NAO	-1.64	1.14	-0.10	-0.08	0.62
P-MT	16.22	20.04	18.23	18.38	0.80
P-RD	253.30	536.40	401.38	405.50	5028.84
P-NAO	-1.83	1.03	-0.73	-0.80	0.51
H-SNOW	0.00	19.00	9.58	12.00	37.82
H-FROST	29.00	69.00	47.42	49.00	129.09
H-NAO	-1.92	1.82	0.28	0.08	1.22
L-MT1	17.53	22.25	19.16	19.00	0.87
L-RD1	181.30	596.40	310.94	296.40	7939.59
L-NAO1	-1.80	1.83	-0.56	-0.53	0.68
L-MT2	17.53	22.25	19.22	19.20	0.87
L-RD2	181.30	596.40	306.34	293.70	7828.61
L-NAO2	-1.80	0.30	-0.65	-0.53	0.38
H-FROST1	29.00	69.00	48.37	50.00	116.02
H-SNOW1	0.00	19.00	10.37	13.00	33.92
H-NAO1	-2.36	1.82	0.11	0.07	1.53
H-FROST2	27.00	69.00	48.26	50.00	120.30
H-SNOW2	0.00	19.00	10.74	13.00	30.19
H-NAO2	-2.36	2.59	0.15	0.07	1.72

[a]For a comprehensive list of these weather covariates see SF5

S.F. 5: Names and definitions of the weather factors

Model	Weather factors name	Weather factors definition	Time of the year
Survival	R-MT	Mean temperature during reproductive period	March to May
	R-RD	Cumulative rainfall during reproductive period	
	R-NAO	North Atlantic Oscillation during reproductive period	
	P-MT	Mean temperature during post-reproductive period	June to October
	P-RD	Cumulative rainfall during post-reproductive period	
	P-NAO	North Atlantic Oscillation during post-reproductive period	
	H-FROST	Number of days with frost during hibernation	November to February
	H-SNOW	Number of days with snow during hibernation	
	H-NAO	North Atlantic Oscillation during hibernation	
Recruitment	L-TM1(2)	Mean temperature during larval development, one (two) year(s) before maturity	May to August
	L-RD1(2)	Cumulative rainfall during larval development, one (two) year(s) before maturity	
	L-NAO1(2)	North Atlantic Oscillation during larval development, one (two) year(s) before maturity	
	H-FROST1(2)	Number of days with frost during hibernation, one (two) year(s) before maturity	November to February
	H-SNOW1(2)	Number of days with snow during hibernation, one (two) year(s) before maturity	
	H-NAO1(2)	North Atlantic Oscillation during hibernation, one (two) year(s) before maturity	

S.F. 6: Survival Model abbreviations and definitions

6.1: States abbreviations and their corresponding definitions

States name	States definition
RSMi	Resident small male immature
RSMm	Resident small male mature
RIMi	Resident intermediate male immature
RIMm	Resident intermediate male mature
RLMm	Resident large male mature
RSFi	Resident small female immature
RSFm	Resident small female mature
RIFi	Resident intermediate female immature
RIFm	Resident intermediate female mature
RLFm	Resident large female mature
TSMi	Transient small male immature
TSMm	Transient small male mature
TIMi	Transient intermediate male immature
TIMm	Transient intermediate male mature
TLMm	Transient large male mature
TSFi	Transient small female immature
TSFm	Transient small female mature
TIFi	Transient intermediate female immature
TIFm	Transient intermediate female mature
TLFm	Transient large female mature

6.2: Events abbreviations and their corresponding definitions

Events name	Events definition
Si	Small immature
SMm	Small male mature
Ii	Intermediate immature
IMm	Intermediate male mature
LMm	Large male mature
SFm	Small female mature
IFm	Intermediate female mature
LFm	Large female mature

S.F. ***7***. Model selection procedure for survival. r = model rank, Dev. = residual deviance, AICc = Akaike information criterion adjusted for small samples, np = number of parameters. Model parameters vary between capture sessions (T), size (SVL), gender (SEX), maturity status (MAT), weather covariates (e.g. R-MT) or are constant (.). For a comprehensive list of these weather covariates see S.F. 5.

r	Model	*Dev.*	*AICc*	*np*
1	δ(T)Φ(SVLxSEX+T)p(SEX+T)	16903.27	17093.17	93
2	δ(T)Φ(SVL+T)p(SEX+T)	16913.76	17093.26	88
3	δ(T)Φ(SVLxSEX+T)p(MAT+T)	16907.69	17095.51	92
4	δ(T)Φ(SVLxSEX+T)p(SVLxSEX+T)	16900.95	17101.28	98
5	δ(T)Φ(SEX+T)p(SEX+T)	16925.57	17105.06	88
6	δ(T)Φ(SVLxSEX+T)p(SVLxMAT+T)	16912.64	17106.72	95
7	δ(T)Φ(T)p(SEX+T)	16933.43	17108.77	86
8	δ(T)Φ(SVLxSEX+T)p(SVL+T)	16921.56	17111.46	93
9	δ(T)Φ(SVLxMAT+T)p(SEX+T)	16928.35	17112.01	90
10	δ(T)Φ(MAT+T)p(SEX+T)	16938.57	17115.99	87
11	RT(T)Φ(SVLxSEX+T)p(SVL)	16969.39	17121.92	75
12	δ(T)Φ(SVLXSEX+T)p(T)	16938.71	17124.45	91
13	δ(T)Φ(SVLxSEX+T)p(SVLxMAT)	16967.04	17123.72	77
14	δ(T)Φ(SVLxSEX+T)p(SEX)	16971.75	17124.28	75
15	δ(T)Φ(SVLxSEX+T)p(MAT)	16974.32	17124.79	74
16	δ(T)Φ(SVLxSEX+T)p(SVLxSEX)	16962.57	17125.46	80
17	δ(T)Φ(SVLxSEX+T)p(.)	16982.97	17131.38	73
18	δ(T)Φ(SVL+P-NAO)p(SEX+T)	17042.54	17186.81	71
19	δ(T)Φ(SVL+H-FROST)p(SEX+T)	17051.78	17196.05	71
20	δ(T)Φ(SVL+R-MT)p(SEX+T)	17057.59	17201.86	71
21	δ(T)Φ(SVLxMAT)p(SEX+T)	17057.92	17204.25	72
22	δ(T)Φ(SVL+H-NAO)p(SEX+T)	17061.48	17205.75	71
23	δ(T)Φ(SVL+P-MT)p(SEX+T)	17063.65	17207.92	71
24	δ(T)Φ(SVLxSEX)p(SEX+T)	17056.52	17209.06	75
25	δ(T)Φ(SVL+R-RD)p(SEX+T)	17065.25	17209.52	71
26	δ(T)Φ(SVL)p(SEX+T)	17067.36	17209.57	70
27	δ(T)Φ(SVL+P-RD)p(SEX+T)	17065.62	17209.89	71
28	δ(T)Φ(SVL+R-NAO)p(SEX+T)	17066.41	17210.69	71
29	δ(T)Φ(SVL+H-SNOW)p(SEX+T)	17067.35	17211.62	71
30	δ(T)Φ(MAT)p(SEX+T)	17080.18	17220.32	69
31	δ(T)Φ(SEX)p(SEX+T)	17080.16	17222.37	70
32	δ(T)Φ(.)p(SEX+T)	17089.05	17227.14	68
33	δ(.)Φ(SVLxSEX+T)p(SEX+T)	17198.76	17349.22	74

S.F. 8. Impact of weather covariates on the survival model: p-values for the F-Test resulting from the ANODEV analysis. Model parameters vary between capture sessions (T), size (SVL), gender (SEX), maturity status (MAT), weather covariates (e.g. R-MT) or are constant (.). For a comprehensive list of these weather covariates see S.F. 5.

Model	F-statistic	p-value
δ(T)Φ(SVL+P-NAO)p(SEX+T)	3.277	0.088
δ(T)Φ(SVL+H-FROST)p(SEX+T)	1.920	0.184
δ(T)Φ(SVL+R-MT)p(SEX+T)	1.155	0.297
δ(T)Φ(SVL+H-NAO)p(SEX+T)	0.677	0.422
δ(T)Φ(SVL+P-MT)p(SEX+T)	0.421	0.525
δ(T)Φ(SVL+R-RD)p(SEX+T)	0.237	0.632
δ(T)Φ(SVL+P-RD)p(SEX+T)	0.195	0.664
δ(T)Φ(SVL+R-NAO)p(SEX+T)	0.106	0.748
δ(T)Φ(SVL+H-SNOW)p(SEX+T)	0.001	0.969

S.F. ***9***. Model selection procedure for recruitment: r = model rank, Dev. = residual deviance, AICc = Akaike information criterion adjusted for small samples, np = number of parameters. Model parameters vary between capture sessions (T), gender (SEX), weather covariates (e.g. RH-NAO1) or are constant (.). For a comprehensive list of these weather covariates see S.F. 5.

r	Model	Deviance	AICc	np
1	δ(.)r(T)p(SEX)	3313.81	3358.15	22
2	δ(.)r(SEX+T)p(SEX)	3313.39	3359.76	23
3	δ(SEX)r(SEX+T)p(SEX)	3313.33	3361.73	24
4	δ(SEX+T)r(SEX+T)p(SEX)	3292.15	3377.36	42
5	δ(SEX+T)r(SEX+T)p(.)	3295.76	3378.92	41
6	δ(SEX+T)r(SEX+T)p(SEX+T)	3259.84	3382.31	60
7	δ(T)r(SEX+T)p(SEX)	3298.38	3381.53	41
8	δ(SEX+T)r(SEX+T)p(T)	3270.67	3391.06	59
9	δ(.)r(H-NAO1)p(SEX)	3449.48	3459.50	5
10	δ(.)r(L-MT2)p(SEX)	3495.25	3505.27	5
11	δ(.)r(L-MT1)p(SEX)	3554.47	3564.49	5
12	δ(.)r(H-SNOW1)p(SEX)	3555.00	3565.02	5
13	δ(.)r(L-RD1)p(SEX)	3566.81	3576.83	5
14	δ(.)r(L-NAO1)p(SEX)	3570.00	3580.02	5
15	δ(.)r(H-FROST1)p(SEX)	3570.96	3580.98	5
16	δ(.)r(L-NAO2)p(SEX)	3572.63	3582.65	5
17	δ(.)r(H-SNOW2)p(SEX)	3572.84	3582.86	5
18	δ(.)r(.)p(SEX)	3574.94	3582.96	4
19	δ(.)r(L-RD2)p(SEX)	3574.18	3584.20	5
20	δ(.)r(SEX)p(SEX)	3574.29	3584.31	5
21	δ(.)r(H-FROST1)p(SEX)	3574.47	3584.49	5
22	δ(.)r(H-NAO2)p(SEX)	3574.83	3584.85	5

S.F. 10. Impact of the weather covariates on the recruitment model: p-values of the F-Test resulting from the ANODEV analysis. Model parameters vary between capture sessions (T), gender (SEX), weather covariates (e.g. R-MT) or are constant (.). For a comprehensive list of these weather covariates see S.F. 5.

r	Model	F-statistic	p-value
9	δ(.)r(H-NAO1)p(SEX)	15.720	0.001
10	δ(.)r(L-MT2)p(SEX)	7.467	0.014
11	δ(.)r(L-MT1)p(SEX)	1.446	0.246
12	δ(.)r(H-SNOW1)p(SEX)	1.405	0.252
13	δ(.)r(L-RD1)p(SEX)	0.547	0.470
14	δ(.)r(L-NAO1)p(SEX)	0.328	0.574
15	δ(.)r(H-FROST1)p(SEX)	0.264	0.614
16	δ(.)r(L-NAO2)p(SEX)	0.152	0.702
17	δ(.)r(H-SNOW2)p(SEX)	0.1380	0.715
19	δ(.)r(L-RD2)p(SEX)	0.050	0.826
21	δ(.)r(H-FROST1)p(SEX)	0.031	0.863
22	δ(.)r(H-NAO2)p(SEX)	0.007	0.934
